# Combined effects of body composition and ageing on joint torque, muscle activation and co-contraction in sedentary women

**DOI:** 10.1007/s11357-014-9652-1

**Published:** 2014-04-19

**Authors:** D. J. Tomlinson, R. M. Erskine, C. I. Morse, K. Winwood, G. L. Onambélé-Pearson

**Affiliations:** 1 Institute for Performance Research, Department of Exercise and Sport Science, Manchester Metropolitan University, Crewe Green Road, Crewe, CW1 5DU UK; 2 Present Address: Research Institute for Sport and Exercise Sciences, Liverpool John Moores University, Liverpool, UK

**Keywords:** Activation, Adiposity, Ageing, Lean mass, Muscle strength, Obesity

## Abstract

This study aimed to establish the interplay between body mass, adiposity, ageing and determinants of skeletal muscle strength. One hundred and two untrained healthy women categorised by age into young (Y) (mean ± SD, 26.7 ± 9.4 years) vs. old (O) (65.1 ± 7.2 years) were assessed for body fat, lean mass, plantar flexion and dorsiflexion maximum voluntary isometric contraction (MVC) torque, muscle activation capacity and antagonist muscle co-contraction. MVC torque normalised to body mass in the obese group was 35 and 29 % lower (*p* < 0.05) in Y and 34 and 31 % lower (*p* < 0.05) in O, compared with underweight and normal weight individuals, respectively. Y with ≥40 % body fat had significantly lower activation than Y with <40 % body fat (88.3 vs. 94.4 %, *p* < 0.05), but O did not exhibit this effect. Co-contraction was affected by ageing (16.1 % in O vs. 13.8 % in Y, *p* < 0.05) but not body composition. There were significant associations between markers of body composition, age, strength and activation capacity, with the strongest correlation between muscle strength and total body mass (*r* ^2^ = 0.508 in Y, *p* < 0.001, vs. *r* ^2^ = 0.204 in O, *p* < 0.01). Furthermore, the age-related loss in plantar flexion (PF) MVC torque was exacerbated in obese compared to underweight, normal weight and overweight individuals (−0.96 vs. −0.54, −0.57 and −0.57 % per year, *p* < 0.05). The negative impact of adiposity on muscle performance is associated with not only muscular but also neural factors. Overall, the effects of ageing and obesity on this system are somewhat cumulative.

## Introduction

Obesity is associated with high body fat and several co-morbidities including lowered functional mobility, particularly in the elderly (Zoico et al. 2004). The latter effect is likely linked to decreased strength to body mass ratio in obese compared with normal weight individuals in both young (Maffiuletti et al. 2007) and aged populations (Rolland et al. 2004). Contributors to strength, over and above muscle tissue content (Erskine et al. 2010a), are agonist muscle activation (Morse et al. 2004), antagonist co-contraction (Klein et al. 2001) and tendon moment arm (Erskine et al. 2010a). Yet, the link between obesity and the principal factors contributing to decreased strength to body mass ratio in both young and ageing populations has yet to be explored. The combined impact of ageing and obesity is of particular interest, as strength is known to decline with ageing (Morse et al. 2005), yet it remains to be seen what effect, if any, increased adiposity has on the ageing-related sarcopenia and in particular on the neuromuscular components of muscle strength.

It could be hypothesised that obesity in both young and old persons would load the antigravity muscles causing hypertrophy, in a manner similar to being placed in a hypergravity environment (Maffiuletti et al. 2007, 2013; Blimkie et al. 1990). Indeed, 3 weeks of simulated hypergravity in young athletes wearing a weighted vest ranging from 7 to 13 % of body weight from morning until night increased participants’ muscle power (Bosco et al. 1984, 1986; Bosco 1985). Interestingly, similar increases in muscle power were demonstrated in postmenopausal women who utilised weighted vests (Klentrou et al. 2007). When relating this to an obese individual who has carried excess weight continuously for years, not weeks, strength gains may follow those seen with resistance training, thus having both neural and muscular underpinnings (Erskine et al. 2010b). Although the loading intensity of increased adiposity is not as high in the conventional resistance training regimes, the volume of loading is likely to be higher with lower loads being lifted during ‘repetitions’ of daily tasks and over longer periods of time. In support of this hypothesis, low-load, high-volume resistance exercise has been shown to stimulate muscle protein synthesis more than traditional high-load, low-volume exercise (Burd et al. 2010).

In terms of the muscular factors of decreased strength, muscle strength is 24.2 and 22.2 % higher (absolute torque or torque normalised to thigh muscle mass, respectively) in obese compared to non-obese adolescent boys (Abdelmoula et al. 2012). In contrast, lower limb maximum voluntary contraction (MVC) torque and power (both absolute and normalised to muscle volume) is lower in obese compared to non-obese persons (Hilton et al. 2008). In terms of the neural factors of decreased strength, one limiting factor could be the central drive. However, studies report that increased adiposity is in fact associated with increased neural sympathetic drive (Alvarez et al. 2002). This would therefore suggest that the deleterious effects of weakness associated with high fat mass are muscular but not neural in origin. However, a study reported a decrease in the muscle activation capacity (85.1 vs. 95.3 %) in obese compared to non-obese adolescent males (Blimkie et al. 1990). Unfortunately, exercise training status was not monitored in this study, and this could account for differences in muscle activation (Hakkinen et al. 1998). In another study, it was found that obese individuals had greater fat-free mass yet similar strength values compared to their normal weight counterparts (Rolland et al. 2004), hence further supporting the suggestion of obesity-related weakness having a neural basis (e.g. decreased agonist activation capacity). Moreover, whilst the expectation is that with increased age, there will be a decrease in agonist activation capacity even in the absence of obesity (Morse et al. 2004), it is unclear whether the above-described decreased activation capacity seen in the adolescents would be mirrored in adults and/or exacerbated in the elderly.

Indeed, skeletal muscle ageing has been well documented and is characterised by lower muscle strength (Morse et al. 2005; Onambele et al. 2006a, b), decreased muscle volume (Thom et al. 2005), decreased agonist activation (Morse et al. 2004) and increased antagonist co-contraction (Klein et al. 2001). The consensus is that many of these ageing effects are caused through decreased habitual physical activity levels and sarcopenia (Rosenberg 1997). Hence, since there is a recognised age-related prevalence in increased body fat percentage termed sarcopenic obesity (Zamboni et al. 2008), characterised by a combination of reduced skeletal muscle mass and increased intramuscular fat (Hilton et al. 2008), obesity would further compound the ageing effects. In support of this hypothesis, sarcopenic obesity would be expected to aggravate the impact of ageing on physical functions including stair climbing, rising from a chair and lifting objects (Rolland et al. 2009). However, Rolland and colleagues did not quantify either agonist muscle activation capacity or antagonist co-contraction (Rolland et al. 2004), thus potentially masking the true impact of sarcopenic obesity.

The present study therefore aimed to contrast how different levels of BMI vs. lean muscle mass vs. adiposity impact on both muscular (absolute and normalised MVC ankle joint torque) and neural factors (agonist muscle activation, antagonist co-contraction) underlying skeletal muscle function. The study also aimed to determine whether the effects of ageing and adiposity were additive. It was hypothesised that: (1) absolute torque in both young and old obese individuals would be higher, but torque relative to body mass lower, compared to underweight, normal weight and overweight individuals; (2) muscle activation would be significantly lower in obese young and old individuals; and (3) the slope of the relationship between adiposity and joint torque, or activation capacity, would be steeper in the older individuals relative to their younger counterparts.

## Method

### Participants

Untrained females (*n* = 102) categorised by age into young (Y) 18–49 years old or old (O) 50–80 years old volunteered to take part in this study. Participants were sub-categorised into four body mass index classifications (BMI—body mass (kg)/stature^2^ (m)) into underweight (BMI <20), normal (BMI 20–24.9), overweight (BMI 25–29.9) and obese (BMI >30). Group information on age, stature and body mass is presented in Table 1. Participants were excluded if there was any issue with lower limb muscles/joints affecting their mobility or ability to exert maximum force. Fasted blood glucose levels were used as an indication of undisclosed peripheral neuropathy, a condition which has a detrimental effect on force production (Hilton et al. 2008).

**Table 1:** Descriptive variables for BMI classifications in both young and old age classifications. The A/G ratio (i.e. android/gynoid dimensional comparison or waist-hip ratio), where it is >1.00, is utilised as an indicator of increased risk of cardiovascular disease (Folsom et al. 2000)

Young (18–49)	Underweight (*n* = 13)	Normal (*n* = 13)	Overweight (*n* = 10)	Obese (*n* = 18)	BMI effect	Ageing effect
Age (years)	23.0 (6.7)	23.2 (7.9)	23.6 (8.0)	30.9 (10.7)	*p* = 0.002	*p* = 0.001
Height (cm)	167.5 (4.7)	164.1 (8.6)	162.8 (7.4)	166.5 (7.6)	*p* = 0.422	*p* = 0.002
Body mass (kg)	52.7 (3.9)	58.3 (6.5)	74.6 (8.3)	97.5 (13.1)	*p* < 0.001	*p* = 0.683
BMI (kg/m^2^)	18.8 (0.9)	21.6 (1.1)	28.1 (2.4)	35.2 (4.4)	*p* < 0.001	*p* = 0.625
Body fat (%)	26.5 (3.9)	30.4 (3.5)	38.7 (5.9)	45.3 (3.9)	*p* < 0.001	*p* = 0.001
Total body fat (kg)	13.7 (2.2)	17.2 (2.7)	28.5 (6.8)	43.2 (7.3)	*p* < 0.001	*p* = 0.376
Total lean mass (kg)	35.7 (3.4)	37.2 (4.7)	42.0 (4.3)	49.4 (7.0)	*p* < 0.001	*p* = 0.002
Fat mass leg (kg)	3.2 (0.5)	3.8 (0.6)	5.8 (1.8)	7.7 (1.5)	*p* < 0.001	*p* = 0.859
A/G ratio	0.70 (0.09)	0.77 (0.12)	0.95 (0.14)	1.06 (0.08)	*p* < 0.001	*p* = 0.062
Old (50–78)	Underweight (*n* = 4)	Normal (*n* = 15)	Overweight (*n* = 18)	Obese (*n* = 11)	BMI effect	Ageing effect
Age (years)	63.8 (5.7)	63.5 (7.7)	68.2 (4.8)	62.5 (9.0)	*p* = 0.183	*p* = 0.001
Height (cm)	159.1 (5.3)	159.4 (5.2)	162.1 (3.8)	162.5 (5.7)	*p* = 0.264	*p* = 0.002
Body mass (kg)	48.4 (4.2)	56.6 (4.3)	71.6 (4.5)	90.1 (16.4)	*p* < 0.001	*p* = 0.683
BMI (kg/m^2^)	19.1 (0.8)	22.2 (1.0)	27.3 (1.2)	34.1 (5.7)	*p* < 0.001	*p* = 0.625
Body fat (%)	26.5 (2.1)	36.0 (3.6)	42.9 (3.3)	46.1 (5.0)	*p* < 0.001	*p* = 0.001
Total body fat (kg)	12.5 (2.0)	19.9 (2.9)	29.8 (3.4)	40.9 (11.3)	*p* < 0.001	*p* = 0.376
Total lean mass (kg)	32.8 (2.4)	33.3 (2.4)	37.4 (2.4)	44.7 (6.7)	*p* < 0.001	*p* = 0.002
Fat mass leg (kg)	2.8 (0.3)	3.8 (0.7)	5.6 (0.1)	6.5 (2.0)	*p* < 0.001	*p* = 0.859
A/G ratio	0.66 (0.09)	0.89 (0.16)	0.97 (0.11)	1.10 (0.08)	*p* < 0.001	*p* = 0.062

Data are presented as mean ± SD

Ethical approval was obtained from the local ethics committee, and all participants gave their written informed consent prior to undertaking any assessment.

### Body composition measure

Body composition analysis (body content of fat, lean muscle and bone) was performed using a dual-energy X-ray absorptiometry (DEXA) scanner (Hologic Discovery: Vertec Scientific Ltd, UK) with participants fasted for 12 h prior to scanning. Participants were laid in a supine position throughout the 7-min scanning procedure. Segmental analysis of the whole body scan provided quantification for lean leg mass, later used in the normalisation of the joint torque data. The android to gynoid ratio (A/G) of each participant was calculated using the Hologic APEX software (version 3.3). The android region was classified as the area between the mid-point of the lumbar spine to the top of the pelvis, whilst the gynoid region was classified as the area between the head of the femur and mid-thigh.

### Muscle strength

Plantar flexion (PF) and dorsiflexion (DF) MVC torque was assessed in the dominant limb using an isokinetic dynamometer (Cybex Norm, Cybex International, New York, NY, USA). Participants were seated with a hip angle of 85° and their dominant leg fully extended. The dominant foot was secured to the footplate of the dynamometer using inextensible straps, ensuring the lateral malleolus was aligned with the centre of rotation. Participants were firmly strapped at the hip, distal thigh and chest with inextensible straps to minimise movement. Prior to undertaking any MVCs, the participants completed a series of warm-up PF and DF contractions.

Participants subsequently performed four isometric PF (×2) and DF (×2) MVCs with the ankle positioned at 0° (anatomically neutral), with 1–2 min of rest between efforts. MVCs were repeated if there was >10 % difference between MVCs to ensure true MVC was obtained. The highest recorded PF and DF MVCs were used for subsequent analysis. Surface electromyography (EMG) of the tibialis anterior (TA) was recorded during all MVCs to calculate antagonist muscle co-contraction during PF MVC (see below for details). Verbal encouragement and biofeedback were provided during each effort.

### Antagonist muscle co-contraction

EMG (using pre-gelled unipolar Ag-AgCl electrodes (Medicost, Denmark)) was used to assess muscle co-contraction of the tibialis anterior during PF MVC. Two electrodes (skin contact size 30 mm × 22 mm) were placed proximally at one third of the tibialis anterior muscle length, mid muscle belly, with a 1–2-mm gap separating each electrode. A reference electrode (Medicost, Denmark) was placed on the head of the fibula. Raw EMG was then recorded at 2,000 Hz, with the band pass filter set at 10–500 Hz and notch at 50 Hz.

Muscle co-contraction (%) was calculated at 0° ankle angle utilising the raw EMG signal (computed as root mean square (RMS) 500 ms either side of the instantaneous peak torque) of the tibialis anterior during PF MVC divided by EMG during DF MVC. Co-contraction torque (Nm) was the product of percent co-contraction and maximal DF torque.

Hence, net PF MVC torque was calculated as the sum of observed maximal PF torque and co-contraction torque. This method assumes that the DF EMG/torque relationship is linear (Maganaris et al. 1998).

### Muscle activation

PF agonist muscle activation was estimated using the interpolated twitch technique (Morse et al. 2004; Pearson and Onambele 2006). Briefly, percutaneous stimuli (DSV Digitimer Stimulator, Digitimer, Herts., UK) were applied to the gastrocnemius using rubber stimulation pads (50 mm × 100 mm; American Imex, Irvine, CA, USA). The two stimulation pads were placed transversely distal to the popliteal crease and myotendinous junction of the soleus. The amplitude of the stimuli was determined prior to interpolation whilst the participant was in a relaxed state; administering twitches starting from 50 mA, with subsequent increments of 50–100 mA, until no further increase in twitch torque was elicited. The assessed supramaximal doublets (i.e. the stimulus intensity above which no further increase in doublet torque was observed with increased stimulus intensity) were superimposed during a maximal PF MVC. The calculation used to establish muscle activation (%) is shown below:

$$ \left(\mathrm{Superimposed}\ \mathrm{doublet}\ \mathrm{torque}/\mathrm{resting}\ \mathrm{doublet}\ \mathrm{torque}\right)\times 100=\mathrm{muscle}\ \mathrm{activation}\left(\%\right) $$

### Statistical analyses

Statistical analyses were carried out using SPSS (version 19, SPSS Inc., Chicago, IL, USA). To determine parametricity, Kolmogorov-Smirnov or Shapiro-Wilk (normal distribution) and Levene’s tests (homogeneity of variance) were utilised. If parametric assumptions were met, a factorial 2 × 4 ANOVA (age × BMI) was utilised with post hoc Bonferroni correction for pairwise comparisons. Where parametric assumptions were breached, Mann-Whitney *U* or Kruskal-Wallis *H* was utilised. Pearson correlations described the relationships between PF MVC and leg lean mass, muscle activation, body mass, fat mass, total lean mass, body fat%, BMI and A/G ratio. Additionally, linear and multiple regressions were used to determine the best predictors of PF MVC. A comparison of the regression coefficients and slopes was conducted using *z* transformations and Student’s *t* statistic. Data are reported as mean ± SD, and statistical significance was accepted when *p* ≤ 0.05. Study power (*β*) and effect size (pε^2^) are also reported.

## Results

### Body composition

Table 1 displays the descriptive values for age, height, body mass, BMI, body fat%, total body fat, total lean mass, leg fat mass and A/G ratio for Y and O females categorised by BMI. A 2 × 4 factorial ANOVA of body fat% revealed a main effect for age (*p* = 0.001; pε^2^ = 0.104; *β* = 0.904) and for BMI (*p* < 0.001; pε^2^ = 0.736; *β* = 1.000) and an age × BMI interaction (*p* = 0.029; pε^2^ = 0.091; *β* = 0.713).

A Mann-Whitney test on leg lean mass revealed a main effect of age (*p* < 0.001), whilst a Kruskal-Wallis test revealed between-group differences for leg lean mass between classifications in Y (*p* < 0.001) and O (*p* < 0.001). However, Y obese had 28 and 27 % more leg lean mass than Y underweight (*p* < 0.001) and Y normal weight (*p* < 0.001) individuals, respectively, whilst obese O had 26, 27 and 20 % more leg lean mass than O underweight (*p* = 0.018), O normal weight (*p* < 0.001) and O overweight (*p* = 0.04), respectively (Table 4).

There were strong positive correlations (*p* < 0.001) between leg lean mass and body mass, and lean mass and body fat, in both Y and O groups (Tables 2 and 3). Ageing affected neither the degree of association in these correlations (*p* > 0.05) nor the slope of the regressions (*p* < 0.05, Table 3). In predicting leg lean mass, a stepwise multiple linear regression was conducted with variables body fat%, body mass, lean mass, fat mass and android to gynoid ratio in all individuals. Total lean mass and body fat% were the only predictors in a stable model, which, in combination, explained 93 % of the leg lean mass in both Y and O individuals (*p* < 0.001; *r* = 0.966).

**Table 2:** Linear regressions (*r* ^2^) between net PF torque, leg lean mass and agonist muscle activation against a series of descriptive variables in young and old untrained females

	Young (*n* = 54)			Old (*n* = 48)		
	PF MVC	Leg lean mass	Activation	PF MVC	Leg lean mass	Activation
Leg lean mass	0.623***	–	0.179**	0.26***	–	NS
Body mass	0.508***	0.749***	0.164**	0.204**	0.677***	NS
Fat mass	0.385***	0.538***	0.157**	0.135*	0.472***	NS
Lean mass	0.600***	0.936***	0.125**	0.242***	0.904***	NS
Body fat%	0.203**	0.240***	0.103*	NS	0.135*	NS
BMI	0.411***	0.548***	0.179**	0.157**	0.559***	NS

*p < 0.05; **p < 0.01; ***p < 0.001

**Table 3:** Pearson correlations, *z* transformation of *r* and Student’s *t* statistic between net PF MVC and leg lean mass against a series of descriptive variables in young and old untrained females

	Young			Old			Correlation coefficient	Ageing effect
	*n*	*r* value	slope	*n*	*r* value	slope	*z* transformation of *r*	Student’s *t* statistic
PF MVC vs. leg lean mass	54	0.79***	19.63	48	0.51***	12.48	1.95	1.90
PF MVC vs. body mass	54	0.71***	1.26	48	0.45**	0.75	1.70	1.85
PF MVC vs. fat mass	54	0.62***	1.70	48	0.37*	0.92	1.57	1.70
PF MVC vs. lean mass	54	0.78***	3.70	48	0.49***	2.28	1.93	1.94
Leg FFM vs. BM	54	0.87***	0.06	48	0.83***	0.06	0.45	0.77
Leg FFM vs. FM	54	0.73***	0.08	48	0.69***	0.07	0.35	0.74
Leg FFM vs. lean mass	54	0.97***	0.19	48	0.95***	0.18	0.50	0.44

*p < 0.05; **p < 0.01; ***p < 0.001 (If *z* > 1.96, *p* < 0.05; *z* > 2.58, *p* < 0.01) (Student’s *t* statistic significance if *t* falls outside ±1.96 *p* < 0.05)

### Muscle strength

A 2 × 4 factorial ANOVA on PF MVC torque revealed a main effect for age (*p* < 0.001; pε^2^ = 0.327; *β* = 1.000) and a main effect for BMI (*p* = 0.001; pε^2^ = 0.152; *β* = 0.937), yet there was no significant age × BMI interaction (*p* = 0.676; pε^2^ = 0.016; *β* = 0.151). However, Y obese had 23 and 20 % higher uncorrected PF MVC torque than underweight (*p* < 0.008) and normal weight (*p* = 0.039) individuals, whilst O individuals revealed no significant between-group differences (*p* > 0.05) (Table 4).

**Table 4:** Displays strength data (PF torque/net PF torque corrected for agonist muscle activation and antagonist co-contraction), agonist activation capacity, antagonist co-contraction and leg lean mass in both young and old BMI classifications

	Young				Old				Young BMI effect	Old BMI effect	Ageing effect
	Underweight (*n* = 13)	Normal (*n* = 13)	Overweight (*n* = 10)	Obese (*n* = 18)	Underweight (*n* = 4)	Normal (*n* = 15)	Overweight (*n* = 18)	Obese (*n* = 11)			
PF torque (Nm)											
PF MVC 0°	125.7 (22.3)	131.6 (28.1)	150.6 (34.8)	163.7 (27.7)	94.4 (32.6)	95.5 (17.1)	102.9 (27.0)	118.0 (30.2)	U N/Ob	–	*p* < 0.001
MVC relative to body mass (Nm/kg)	2.38 (0.38)	2.26 (0.45)	2.09 (0.34)	1.67 (0.30)	1.96 (0.67)	1.69 (0.28)	1.44 (0.35)	1.33 (0.37)	UN O/Ob	U/Ob	*p* < 0.001
Net PF torque (Nm)											
PF MVC 0°	134.9 (21.4)	142.1 (25.7)	165.8 (33.3)	185.4 (36.8)	105.6 (28.1)	109.6 (17.4)	122.6 (26.9)	134.7 (31.9)	U N/Ob	–	*p* < 0.001
MVC relative to body mass (Nm/kg)	2.56 (0.38)	2.45 (0.41)	2.27 (0.27)	1.90 (0.30)	2.03 (0.40)	1.99 (0.39)	1.71 (0.35)	1.52 (0.39)	U N/Ob	N/Ob	*p* < 0.001
DF MVC 0° (Nm)	22.8 (7.2)	25.3 (5.0)	27.0 (7.2)	26.9 (7.0)	20.1 (4.1)	19.6 (3.9)	22.6 (5.0)	28.4 (6.3)	–	U N O/Ob	*p* = 0.025
Activation (%)	95.0 (5.0)	93.9 (7.8)	94.1 (6.3)	87.9 (10.4)	90.1 (12.1)	86.4 (10.6)	82.2 (13.7)	84.0 (15.2)	–	–	*p* = 0.001
Co-contraction (%)	15.7 (7.2)	17.2 (8.5)	15.2 (5.8)	16.1 (5.9)	15.9 (10.6)	15.1 (8.8)	12.9 (7.9)	12.8 (6.8)	–	–	*p* = 0.042
Leg lean mass (kg)	5.99 (0.78)	6.10 (0.97)	7.31 (1.05)	8.30 (1.35)	5.37 (0.52)	5.26 (0.52)	5.81 (0.64)	7.21 (1.38)	UN/Ob	U N O/Ob	*p* < 0.001

Data are presented as mean ± SD

(*U* underweight, *N* normal weight, *O* overweight, *Ob* obese

PF MVC torque relative to body mass was higher in Y vs. O (*p* < 0.001; pε^2^ = 0.291; *β* = 1.000) and differed according to BMI (*p* < 0.001; pε^2^ = 0.285; *β* = 1.000), but there was no significant age × BMI interaction (*p* = 0.410; pε^2^ = 0.030; *β* = 0.258). However, Y obese had 43, 35 and 25 % lower PF MVC torque relative to body mass than Y underweight (*p* < 0.001), Y normal weight (*p* < 0.001) and Y overweight (*p* = 0.031), respectively, whilst obese O exhibited 43 % lower uncorrected PF MVC torque relative to body mass than O underweight (*p* = 0.032) (Table 4).

The net PF MVC torque revealed a main effect of age (*p* < 0.001; pε^2^ = 0.293; *β* = 1.000) and BMI (*p* < 0.001; pε^2^ = 0.224; *β* = 0.995), but no significant age × BMI interaction (*p* = 0.581; pε^2^ = 0.021; *β* = 0.184). However, Y obese had 27 and 23 % higher corrected PF MVC torque than Y underweight (*p* < 0.001) and Y normal weight (*p* = 0.002) individuals, whilst O revealed no significant between-group differences (*p* > 0.05) (Table 4).

Net PF MVC torque/body mass revealed a main effect of age (*p* < 0.001; pε^2^ = 0.281; *β* = 1.000) and BMI (*p* < 0.001; pε^2^ = 0.280; *β* = 1.000), but no significant age × BMI interaction (*p* = 0.821; pε^2^ = 0.010; *β* = 0.107). However, Y obese had 35 and 29 % lower net PF MVC torque/body mass than Y underweight (*p* < 0.001) and Y normal weight (*p* < 0.001) individuals, whilst obese O had 31 % lower net PF MVC torque/body mass than O normal weight (*p* = 0.016) (Table 4).

There were strong positive correlations (*p* < 0.001) between net PF MVC torque and body mass, lean mass and body fat in both Y and O groups (as seen in Table 2). Ageing affected neither the degree of association in these correlations (*p* > 0.05) nor the slope of the regressions (*p* > 0.05, Table 3). In predicting net PF MVC, a stepwise multiple linear regression was conducted with independent variables being body fat%, body mass, lean mass, fat mass and android to gynoid ratio, for the pooled study population (i.e. all Y and O data). Total lean mass was the only predictor in the stable model and explained 51 % of net PF MVC, and body mass normalised torque regardless of age (*p* < 0.001; *r* = 0.710).

**Fig. 1:**
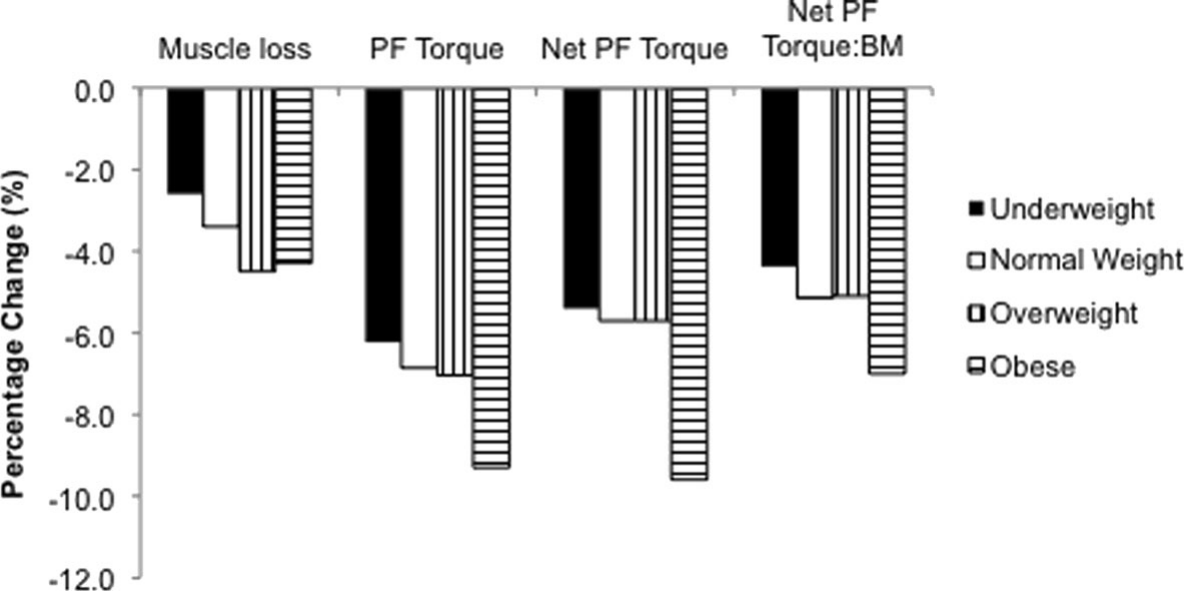
Relative change (mean % change per 10 years assuming percentage change is linear) by BMI class (a) muscle loss, (b) PF torque, (c) net PF torque, and (d) net PF torque normalised for BM

Net PF MVC and leg lean mass were correlated in both the Y (*p* < 0.001; *r* ^2^ = 0.623) and O (*p* < 0.001; *r* ^2^ = 0.260) age groups, with similar slopes in the two age groups (Table 3). Figure 1 displays the percent change per 10 years (assuming % change is linear) in muscle loss, PF torque, net PF torque and net PF torque normalised for BM categorised by BMI.

### Muscle co-contraction

There was no significant difference in antagonist co-contraction between BMI groups (*p* = 0.730) (Table 4). Yet, there was an effect of age, with O exhibiting lower co-activation than Y (16.1 vs. 13.8 %; *p* = 0.042).

### Voluntary muscle activation

**Fig. 2:**
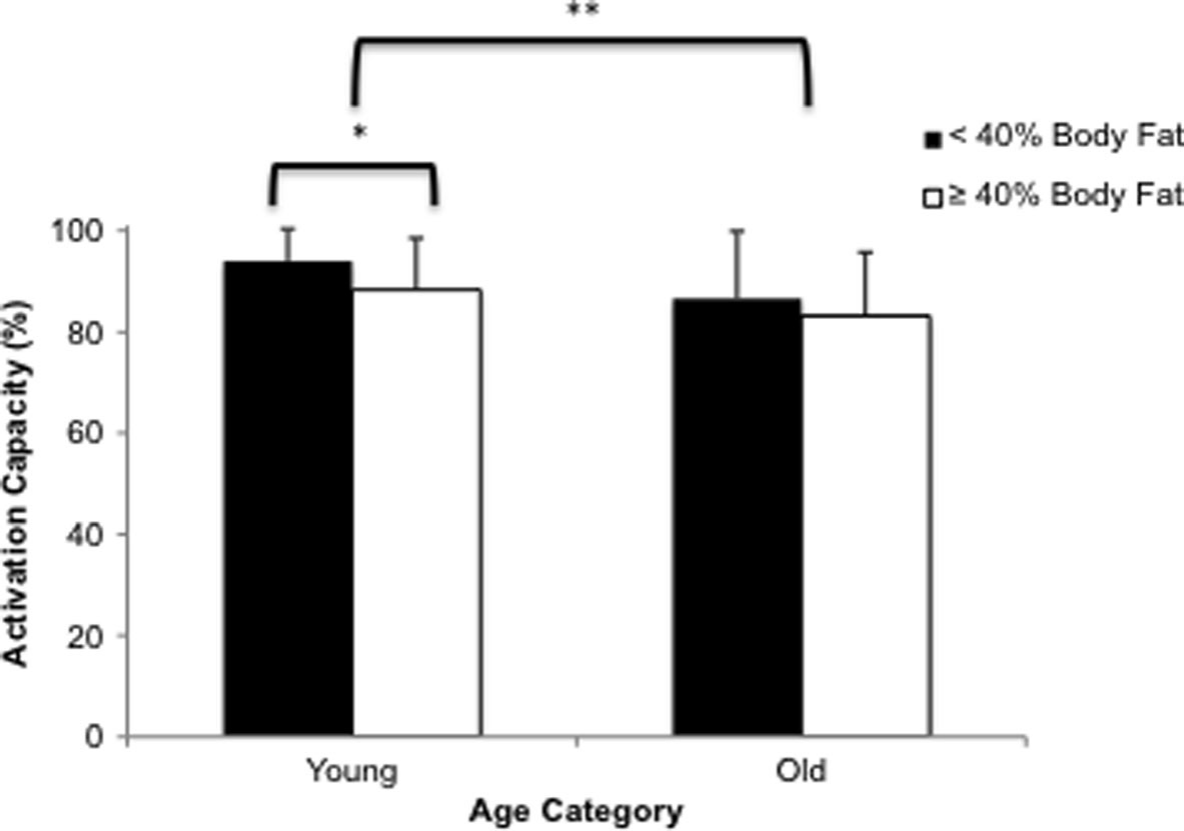
Impact of ageing on PF activation capacity in low body fat (<40 %) and high body fat (>40 %) individuals. The threshold set at ≥40 % body fat as being defined obese is due to previous work in an adult population (Rolland et al. 2004). Data are presented as mean ± SD (*p < 0.05; **p < 0.01)

Muscle activation did not differ between BMI groups in either Y (*p* = 0.138) or O (*p* = 0.701) (Table 4). When Y and O were categorised by body fat% (Fig. 2), muscle activation was higher in Y low body fat% (BF%) than Y high BF% (*p* = 0.019) but there was no difference in O (*p* = 0.458) (Fig. 1). Y also demonstrated higher muscle activation capacity than O (92 vs. 84.5 %; *p* = 0.001).

## Discussion

Our data have provided supportive evidence for our hypothesis that absolute torque in young obese individuals would be higher compared to that of underweight and normal weight individuals. However, the old obese females did not exhibit significantly higher absolute torque compared to the other three BMI classifications. Unexpectedly, when torque was normalised to body mass, the young obese were significantly weaker than the other three BMI classifications, whereas the old obese were only weaker than their old underweight counterparts, thereby suggesting a somewhat protective impact of obesity for the old, or at least, this suggest that the effects of ageing and obesity are not necessarily cumulative where absolute joint torque is concerned. Another partially supported hypothesis was also the observation that high adiposity only decreased activation capacity in the young whereas it had no effect in the old individuals. The summation of a negative impact of obesity to ageing in this population was seen in terms of neuromuscular ageing whereby there were marked differences in the slopes of the relationships between adiposity, muscle torque and activation capacity. The above observations culminated in the fact that with torque normalised for body mass and corrected for activation and co-activation, the obesity-induced weakness was true for all pairwise BMI category comparisons in the young. In the old, however, once torque was normalised for body mass and corrected for activation and co-activation, the only significant BMI pairwise difference was between obese vs. normal weight.

### Body composition

The loading effect of chronically high levels of adiposity, body mass and lean mass appears to provide a stimulus to the antigravity muscles of the lower limb similar to resistance training of increased muscle mass (Erskine et al. 2010b) (as shown in Tables 2, 3 and 4). However, whilst 3 weeks of simulated hypergravity (Bosco 1985) would cause adaptations at a neurogenic level, the mean duration of obesity is longitudinal at 13 years (Abdullah et al. 2011), and hence, adaptations to this condition were not expected to be neural led. At the muscle level, low-load, high-volume resistance exercise has been shown to stimulate muscle protein synthesis more than traditional high-load, low-volume resistance exercise (Burd et al. 2010), and our hypothesis was that obesity would have a similar effect, thus leading to increased muscle mass and strength over time.

Leg lean mass decreased with age in all BMI classifications, but this age-related decrease was exacerbated in the overweight and obese individuals who lost 4.5 and 4.3 %, respectively, compared to 2.6 and 3.4 % in underweight and normal weight individuals every 10 years (data calculated assuming a linear regression, Fig. 1). This blunted effect of loading in the old participants on leg lean mass may possibly be attributed to lower levels of anabolic circulating hormones such as insulin-like growth factor-1 (IGF-I) (Galli et al. 2012) and the increase of catabolic cytokines such as interleukin-6 (IL-6) and tumour necrosis factor α (TNF-α) (Visser et al. 2002; Hotamisligil et al. 1995) seen in older vs. younger individuals.

### Muscle strength

Increased adiposity in the young was previously shown to have a positive effect on the loaded antigravity muscles of the knee extensors in terms of both absolute MVC isometric torque (Hulens et al. 2001; Maffiuletti et al. 2007; Abdelmoula et al. 2012) and isokinetic power (Maffiuletti et al. 2007; Hulens et al. 2002), but not when torque was normalised to body mass, as obese individuals were shown to be weaker (Maffiuletti et al. 2007). Our current results in the plantar flexors mirror these observations.

In the old individuals, there was also a trend of increasing absolute torque with BMI classification, yet there were no significant differences in MVC torque normalised to body mass between BMI classifications (Table 4), except, where the old obese were significantly weaker compared to normal weight individuals. This finding partly supports those of Rolland et al. (2004), who reported greater absolute knee extensor strength in the obese. Additionally, the old obese individuals lost more MVC torque compared to the losses in leg lean mass (27 vs. 13 %); this is comparable to research by Delmonico et al. (2009) who attributed losses in strength to lowering in muscle quality. This may possibly be attributed to intrinsic changes in muscle properties such as selected atrophy of type II fibres (Lexell and Taylor 1991) and/or a decrease in muscle fascicle pennation angle (Morse et al. 2005) normally seen in ageing.

Whilst our old females did not meet the criterion for sarcopenia (i.e. appendicular skeletal muscle mass ÷ height (m^2^) = mean ± SD 9.6 ± 1.5 kg/m^2^ in this group vs. ≤5.67 kg/m^2^ criterion; Newman et al. 2003), they displayed typical ageing-related characteristics of significantly lower isometric MVC torque (−25 %, *p* < 0.001), agonist activation (92.1 vs. 84.5 %, *p* < 0.05) and leg lean mass (7.03 vs. 5.92 kg, *p* < 0.001). Ageing-associated α-motor neuron degeneration and denervation (Brown 1972), higher levels of IL-6 and TNF-α (Visser et al. 2002) may explain some of these effects. Assuming the loss of joint torque is linear, PF MVC torque appeared to be exacerbated in obese individuals who would lose 9.6 % of maximal torque compared to 5.4, 5.7 and 5.7 % in underweight, normal weight and overweight individuals every 10 years (data calculated assuming a linear regression, Fig. 1).

The effect of adiposity per se on muscle strength suggested a positive association with absolute PF MVC in both young and old groups (Table 2). Yet, when factoring total adiposity in a multiple linear regression, lean muscle mass was the only significant predictor of PF MVC torque. Since adiposity is not a contractile tissue, the apparent positive association with muscle strength can only be due to high adiposity being associated with greater muscle mass in our population.

Previous research on obesity in ageing and muscle performance mainly selected the loaded knee extensors as an indicator of lower limb contractile capacity. The work presented in this paper is novel in that (a) it utilised the plantar flexors, a muscle group which has a key functional relevance to older persons due to its documented contribution in the maintenance of postural balance (Onambele et al. 2006a), so much so that approximately 80 % of the variance in postural balance can be attributed to the functional characteristics of this muscle group, and (b) obesity also has been documented as having a detrimental impact on postural balance (Maffiuletti et al. 2005).

### Co-contraction

To our knowledge, this is the first study to describe antagonist muscle co-contraction in an obese population. Our study reports, at face value, a protective effect of obesity during ageing since old obese individuals had significantly lower co-contraction than their younger counterparts (16.1 vs. 13.8 %, *p* < 0.042), thereby potentially contributing to higher agonistic forces and hence better control of joint motion. This was an unexpected finding as ageing is associated with increased co-contraction in the hamstrings (Macaluso et al. 2002) and the triceps surae (Onambele et al. 2006a). However, since joint stabilisation through co-contraction is a strategy used when muscle weakness is present (Hortobagyi and DeVita 2000), the old obese are in fact doubly disadvantaged through being both weaker and less able to co-contract their antagonist muscles compared to age-matched normal weight counterparts, potentially leading to increased risk of joint pathologies (Felson 1995).

### Voluntary muscle activation

Previous work examining muscle strength differences between obese and non-obese individuals did not account for agonist activation and antagonist co-contraction, thus underestimating the true contractile torque potential, even when such work normalised MVC torque to muscle mass (Abdelmoula et al. 2012; Maffiuletti et al. 2007). Our study demonstrates that high levels of adiposity, with the threshold set at ≥40 % body fat (Rolland et al. 2009), have a significantly negative impact on agonist muscle activation in Y (88 vs. 94 %) but not O (83 vs. 87 %) individuals. The Y data supports the Blimkie et al. (1990) study that reported the agonist activation capacity of obese adolescents aged 15–18 years old to be significantly lower than that of non-obese counterparts (85 vs. 95 %). It would therefore appear that unlike simulated hypergravity which enhances muscle activation (Bosco et al. 1986), obesity-mediated chronic overloading has either no (as seen in the O) or a negative (as seen in the Y) impact on voluntary muscle activation. This would tend to refute the suggestion by Bosco et al. (1986) of a neurogenic mechanism for increased muscle power in the obese. We would argue therefore that any strengthening effect of obesity would be through muscular factors such as increased protein synthesis rate (Burd et al. 2010), which is evidenced through the phenotypic expression of greater absolute lean mass in this population.

A limitation of the present study, as with previous work (Blimkie et al. 1990; Maffiuletti et al. 2007; Abdelmoula et al. 2012), is the failure to quantify anatomical cross-sectional area and using instead leg lean mass and estimations of fat-free mass and thigh muscle mass as an indication of agonist muscle size. Not accounting for muscle fascicle pennation angle (and hence sarcomeres in parallel) and fascicle length (and hence sarcomeres in series) limits the ability to explain differences in force and power. Future work should determine a more accurate index of muscle size by measuring the physiological cross-sectional area (muscle volume/fascicle length) (Fukunaga et al. 1996).

## Conclusion

The present study demonstrates that antigravity muscles adapt to chronically different levels of adiposity in both young and elderly individuals. Interestingly, the magnitude of the effect of obesity in terms of both absolute MVC joint torque and torque normalised to leg lean mass appears to be blunted in the older group. Also notable, the rate of ageing (i.e. the slope of deleterious changes in neuromuscular properties) in the BMI sub-categories is most dramatic for the high-adiposity groups.

## References

[CR1] Abdelmoula A, Martin V, Bouchant A, Walrand S, Lavet C, Taillardat M, Maffiuletti NA, Boisseau N, Duche P, Ratel S (2012). Knee extension strength in obese and nonobese male adolescents. Appl Physiol Nutr Metab.

[CR2] Abdullah A, Wolfe R, Stoelwinder JU, de Courten M, Stevenson C, Walls HL, Peeters A (2011). The number of years lived with obesity and the risk of all-cause and cause-specific mortality. Int J Epidemiol.

[CR3] Alvarez GE, Beske SD, Ballard TP, Davy KP (2002). Sympathetic neural activation in visceral obesity. Circulation.

[CR4] Blimkie CJ, Sale DG, Bar-Or O (1990). Voluntary strength, evoked twitch contractile properties and motor unit activation of knee extensors in obese and non-obese adolescent males. Eur J Appl Physiol Occup Physiol.

[CR5] Bosco C (1985). Adaptive response of human skeletal muscle to simulated hypergravity condition. Acta Physiol Scand.

[CR6] Bosco C, Zanon S, Rusko H, Dal Monte A, Bellotti P, Latteri F, Candeloro N, Locatelli E, Azzaro E, Pozzo R (1984). The influence of extra load on the mechanical behavior of skeletal muscle. Eur J Appl Physiol Occup Physiol.

[CR7] Bosco C, Rusko H, Hirvonen J (1986). The effect of extra-load conditioning on muscle performance in athletes. Med Sci Sports Exerc.

[CR8] Brown WF (1972). A method for estimating the number of motor units in thenar muscles and the changes in motor unit count with ageing. J Neurol Neurosurg Psychiatry.

[CR9] Burd NA, West DW, Staples AW, Atherton PJ, Baker JM, Moore DR, Holwerda AM, Parise G, Rennie MJ, Baker SK, Phillips SM (2010). Low-load high volume resistance exercise stimulates muscle protein synthesis more than high-load low volume resistance exercise in young men. PLoS ONE.

[CR10] Delmonico MJ, Harris TB, Visser M, Park SW, Conroy MB, Velasquez-Mieyer P, Boudreau R, Manini TM, Nevitt M, Newman AB, Goodpaster BH (2009). Longitudinal study of muscle strength, quality, and adipose tissue infiltration. Am J Clin Nutr.

[CR11] Erskine RM, Jones DA, Williams AG, Stewart CE, Degens H (2010). Inter-individual variability in the adaptation of human muscle specific tension to progressive resistance training. Eur J Appl Physiol.

[CR12] Erskine RM, Jones DA, Williams AG, Stewart CE, Degens H (2010). Resistance training increases in vivo quadriceps femoris muscle specific tension in young men. Acta Physiol (Oxf).

[CR13] Felson DT (1995). Weight and osteoarthritis. J Rheumatol Suppl.

[CR14] Folsom AR, Kushi LH, Anderson KE, Mink PJ, Olson JE, Hong CP, Sellers TA, Lazovich D, Prineas RJ (2000). Associations of general and abdominal obesity with multiple health outcomes in older women: the Iowa Women’s Health Study. Arch Intern Med.

[CR15] Fukunaga T, Roy RR, Shellock FG, Hodgson JA, Edgerton VR (1996). Specific tension of human plantar flexors and dorsiflexors. J Appl Physiol.

[CR16] Galli G, Pinchera A, Piaggi P, Fierabracci P, Giannetti M, Querci G, Scartabelli G, Manetti L, Ceccarini G, Martinelli S, Di Salvo C, Anselmino M, Bogazzi F, Landi A, Vitti P, Maffei M, Santini F (2012). Serum insulin-like growth factor-1 concentrations are reduced in severely obese women and raise after weight loss induced by laparoscopic adjustable gastric banding. Obes Surg.

[CR17] Hakkinen K, Kallinen M, Izquierdo M, Jokelainen K, Lassila H, Malkia E, Kraemer WJ, Newton RU, Alen M (1998). Changes in agonist–antagonist EMG, muscle CSA, and force during strength training in middle-aged and older people. J Appl Physiol.

[CR18] Hilton TN, Tuttle LJ, Bohnert KL, Mueller MJ, Sinacore DR (2008). Excessive adipose tissue infiltration in skeletal muscle in individuals with obesity, diabetes mellitus, and peripheral neuropathy: association with performance and function. Phys Ther.

[CR19] Hortobagyi T, DeVita P (2000). Muscle pre- and coactivity during downward stepping are associated with leg stiffness in aging. J Electromyogr Kinesiol.

[CR20] Hotamisligil GS, Arner P, Caro JF, Atkinson RL, Spiegelman BM (1995). Increased adipose tissue expression of tumor necrosis factor-alpha in human obesity and insulin resistance. J Clin Invest.

[CR21] Hulens M, Vansant G, Lysens R, Claessens AL, Muls E, Brumagne S (2001). Study of differences in peripheral muscle strength of lean versus obese women: an allometric approach. Int J Obes Relat Metab Disord.

[CR22] Hulens M, Vansant G, Lysens R, Claessens AL, Muls E (2002). Assessment of isokinetic muscle strength in women who are obese. J Orthop Sports Phys Ther.

[CR23] Klein CS, Rice CL, Marsh GD (2001). Normalized force, activation, and coactivation in the arm muscles of young and old men. J Appl Physiol.

[CR24] Klentrou P, Slack J, Roy B, Ladouceur M (2007). Effects of exercise training with weighted vests on bone turnover and isokinetic strength in postmenopausal women. J Aging Phys Act.

[CR25] Lexell J, Taylor CC (1991). Variability in muscle fibre areas in whole human quadriceps muscle: effects of increasing age. J Anat.

[CR26] Macaluso A, Nimmo MA, Foster JE, Cockburn M, McMillan NC, De Vito G (2002). Contractile muscle volume and agonist–antagonist coactivation account for differences in torque between young and older women. Muscle Nerve.

[CR27] Maffiuletti NA, Agosti F, Proietti M, Riva D, Resnik M, Lafortuna CL, Sartorio A (2005). Postural instability of extremely obese individuals improves after a body weight reduction program entailing specific balance training. J Endocrinol Investig.

[CR28] Maffiuletti NA, Jubeau M, Munzinger U, Bizzini M, Agosti F, De Col A, Lafortuna CL, Sartorio A (2007). Differences in quadriceps muscle strength and fatigue between lean and obese subjects. Eur J Appl Physiol.

[CR29] Maffiuletti N, Ratel S, Sartorio A, Martin V (2013). The impact of obesity on in vivo human skeletal muscle function. Curr Obes Rep.

[CR30] Maganaris CN, Baltzopoulos V, Sargeant AJ (1998). Differences in human antagonistic ankle dorsiflexor coactivation between legs; can they explain the moment deficit in the weaker plantarflexor leg?. Exp Physiol.

[CR31] Morse CI, Thom JM, Davis MG, Fox KR, Birch KM, Narici MV (2004). Reduced plantarflexor specific torque in the elderly is associated with a lower activation capacity. Eur J Appl Physiol.

[CR32] Morse CI, Thom JM, Reeves ND, Birch KM, Narici MV (2005). In vivo physiological cross-sectional area and specific force are reduced in the gastrocnemius of elderly men. J Appl Physiol.

[CR33] Newman AB, Kupelian V, Visser M, Simonsick E, Goodpaster B, Nevitt M, Kritchevsky SB, Tylavsky FA, Rubin SM, Harris TB (2003). Sarcopenia: alternative definitions and associations with lower extremity function. J Am Geriatr Soc.

[CR34] Onambele GL, Narici MV, Maganaris CN (2006). Calf muscle-tendon properties and postural balance in old age. J Appl Physiol.

[CR35] Onambele GN, Bruce SA, Woledge RC (2006). Oestrogen status in relation to the early training responses in human thumb adductor muscles. Acta Physiol (Oxf).

[CR36] Pearson SJ, Onambele GN (2006). Influence of time of day on tendon compliance and estimations of voluntary activation levels. Muscle Nerve.

[CR37] Rolland Y, Lauwers-Cances V, Pahor M, Fillaux J, Grandjean H, Vellas B (2004). Muscle strength in obese elderly women: effect of recreational physical activity in a cross-sectional study. Am J Clin Nutr.

[CR38] Rolland Y, Lauwers-Cances V, Cristini C, Abellan van Kan G, Janssen I, Morley JE, Vellas B (2009). Difficulties with physical function associated with obesity, sarcopenia, and sarcopenic-obesity in community-dwelling elderly women: the EPIDOS (EPIDemiologie de l’OSteoporose) study. Am J Clin Nutr.

[CR39] Rosenberg IH (1997). Sarcopenia: origins and clinical relevance. J Nutr.

[CR40] Thom JM, Morse CI, Birch KM, Narici MV (2005). Triceps surae muscle power, volume, and quality in older versus younger healthy men. J Gerontol A Biol Sci Med Sci.

[CR41] Visser M, Pahor M, Taaffe DR, Goodpaster BH, Simonsick EM, Newman AB, Nevitt M, Harris TB (2002). Relationship of interleukin-6 and tumor necrosis factor-alpha with muscle mass and muscle strength in elderly men and women: the Health ABC Study. J Gerontol A Biol Sci Med Sci.

[CR42] Zamboni M, Mazzali G, Fantin F, Rossi A, Di Francesco V (2008). Sarcopenic obesity: a new category of obesity in the elderly. Nutr Metab Cardiovasc Dis.

[CR43] Zoico E, Di Francesco V, Guralnik JM, Mazzali G, Bortolani A, Guariento S, Sergi G, Bosello O, Zamboni M (2004). Physical disability and muscular strength in relation to obesity and different body composition indexes in a sample of healthy elderly women. Int J Obes Relat Metab Disord.

